# Development of an Oriental Medicine Discrimination Method through Analysis of Steroidal Saponins in *Dioscorea nipponica* Makino and Their Anti-Osteosarcoma Effects

**DOI:** 10.3390/molecules24224022

**Published:** 2019-11-06

**Authors:** Joo Tae Hwang, Ki-Sun Park, Jin Ah Ryuk, Hye Jin Kim, Byoung Seob Ko

**Affiliations:** Korea Institute of Oriental Medicine, Daejeon 34054, Korea; jthwang@kiom.re.kr (J.T.H.); kisunpark@kiom.re.kr (K.-S.P.); yukjinah@kiom.re.kr (J.A.R.); kimhyejin43@kiom.re.kr (H.J.K.)

**Keywords:** *Dioscorea nipponica* Makino, steroidal saponin, HPLC-UV, UPLC-QTOF/MS, validation, osteosarcoma, apoptosis

## Abstract

To prevent confusing *Dioscorea nipponica* (DN), an Oriental medicine, with *Dioscorea quinquelobata* (DQ) and *Dioscorea septemloba* (DS), a simple and accurate quantitative analysis method using HPLC combined with ultraviolet (UV) detection was developed and verified with UPLC-QTOF/MS through identification of five saponin glycosides: protodioscin (**1**), protogracillin (**2**), pseudoprotodioscin (**3**), dioscin (**4**), and gracillin (**5**). The newly developed analysis method showed sufficient reproducibility (<1.91%) and accuracy (92.1%–102.6%) and was able to identify DN based on the presence of compound **3** (13.821 ± 0.037 mg/mL) and the absence of **5**. Compound **1**, which is present in DN at a relatively high level (159.983 ± 0.064 mg/mL), was also an important marker for identification. Among the three species, DN showed the strongest activation of apoptotic signaling in osteosarcoma cells, while the four compounds detected in DN showed IC_50_ values of 6.43 (**1**), 10.61 (**2**), 10.48 (**3**), and 6.90 (**4**). In conclusion, the strong inhibitory effect of DN against osteosarcoma was confirmed to be associated with **1** and **4**, which is also related to the quantitative results. Therefore, the results of this study might provide important information for quality control related to Oriental medicine.

Academic Editors: In-Soo Yoon and Hyun-Jong Cho

## 1. Introduction

Among the various *Dioscorea* families, *Dioscorea nipponica* (DN) is a wild perennial species that is widely distributed in the Korean peninsula, Japan, and China, along with *Dioscorea tokoro*, *Dioscorea japonica*, *Dioscorea tenuipes*, *Dioscorea quinquelobata* (DQ), and *Dioscorea septemloba* (DS). DN, the dried roots and stems of which are used for medicinal purposes in Oriental medicine, is known as Cheon-san-ryong. This medicine has traditionally been prescribed for the treatment of rheumatism, asthma, and bronchitis; alleviating pain; and improving blood circulation. Additionally, the origin of the species specified in the standard medicines of North Korea [1] and China [2] is designated as DN. In China, which is a major producer of DN, approximately 49 species of the genus *Dioscorea* are found, and two of these species, DN and *Dioscorea panthaica*, are systematically managed by the Chinese Pharmacopeia Committee (CHD) as raw medicine materials [3]. However, in the private sector in South Korea, this medicine is not explicitly distinguished from DQ and DS, which are homogeneous and morphologically similar to DN [4]. Thus, a scientific and systematic approach to clearly identify the origin of DN is absolutely necessary, but no relevant research has been carried out to date. Additionally, quality control of raw materials is always necessary for commercial development, and the development of chemical analysis methods is key to quality control.

**Figure 1:**
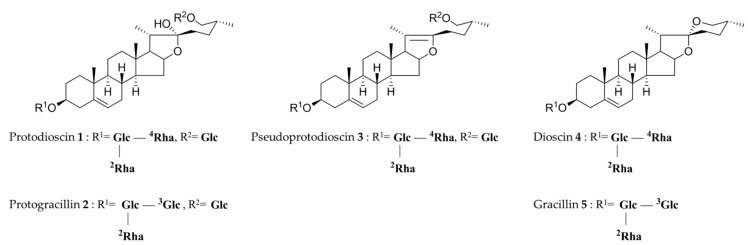
Chemical structures of the standards for HPLC validations (Glc = β-_D_-glucopyranosyl, Rha = α-_L_–rhamnopyranosyl).

As part of this study, previously reported compounds from the *Dioscorea* family, including DQ and DS, were searched. In previous studies, steroidal saponins have mainly been found in members of the genus *Dioscoreaceae*, and the identified active components are steroidal saponins, including furostanol saponins and isospirostanol saponins [5]. Among the identified compounds, the steroidal saponins of three furostanol derivatives, protodioscin (**1**), protogracillin (**2**), and pseudoprotodioscin (**3**), and two spirostanol derivatives, dioscin (**4**), and gracillin (**5**), could be utilized as important indicators for development of an analytical method (Figure 1). Due to their biological activities, members of the genus *Dioscoreaceae* are being studied by numerous researchers to achieve efficient separation and purification of one of the steroids, diosgenin, and its steroidal saponin, dioscin [6]. For example, a recent study reported the optimized extraction of these compounds, with the highest extract content when 50% acetonitrile (ACN) was used for 60 minutes in ultrasonic extraction. However, compared with the yield of individual compounds, protodioscin had the best extraction efficiency at 50% ACN, whereas for dioscin, 70% ACN was more efficient [7]. Several analytical methods for determination of the above compounds have been reported previously. Although these analyses primarily used LC-ESI-MS or MS/MS, the development of analysis methods using HPLC is also frequently reported [8,9,10]. However, these analytical methods could not be fully verified because there were not sufficient indicator components to clearly distinguish the origin of the drug from similar species, and the steroidal saponins of *Dioscorea* have been tentatively confirmed by MS fragmentation analyses. Additionally, in the case of steroidal saponins, ultraviolet (UV) detection is generally less sensitive, and thus, it is natural to use ELSD (evaporative scattering detection) detectors, including for MS analysis, frequently. These detectors can be a powerful detection method but limit analysis options. Therefore, it is also very important to develop an analysis method that is reliable and exhibits sufficient sensitivity using UV detectors for a wide range of sample extract analyses.

Saponins show potential as anticancer agents. As previously reported, wild species of *Dioscorea* families, including DN, were found to contain at least 10 to 20 times more dioscin, a major pharmacological component and a saponin exhibiting strong anticancer activity, than cultivated species, such as *Dioscorea opposita* or *Dioscorea batatas* [11]. This finding shows that DN is a very attractive material for commercialization, which can be realized through further research. Recently, the positive effects of steroidal saponins as a treatment for osteosarcoma, which is known to have a poor prognosis, have been reported. The cause of osteosarcoma is unclear, and in most cases, it appears to be sporadic, especially in people in their teens, where it is associated with increased growth. Currently, surgery and chemotherapy are combined to treat osteosarcoma, and efforts are being made to develop new natural products for effective chemotherapy. Therefore, it is necessary to investigate the possibility of treating osteosarcoma with DS, DQ, and DN that contain steroidal saponins and to examine the most effective extracts [12,13,14,15,16,17,18,19,20].

Thus, here, an accurate and reproducible HPLC/UV analysis method was developed to isolate five steroid saponins that were identified in the *Dioscorea* families. This method was then applied to traditional medicines and allowed differentiation of raw materials originating from similar but different species. Quadrupole time-of-flight MS (QTOF/MS) combined with ultra-performance liquid chromatography (UPLC) was used to confirm the steroidal saponins in the samples. In addition, the bioactive compounds in raw plants were quantified, and the ability of these compounds to inhibit osteosarcoma was tested to identify new pharmacological activities of DN and its active components. 

## 2. Results and Discussion

### 2.1. Optimization of Chromatographic Conditions

Generally, HPLC/UV analysis is not recommended for steroidal saponins because they lack a chromophore suitable for UV detection [3]. This challenge is a good motivator for studying whether the target compounds can be detected accurately without being influenced by impurities; although ELSD [21] or ESI-MS [22,23] detectors have been used in previous studies, low-wavelength UV offers superior sensitivity. The main focus of this study was to develop reliable analysis methods that are more efficient and generally easy for users to analyze rather than to develop powerful but less frequently used and costly analyses using various MS or MS/MS, such as the above mentioned. This is a very important factor in the industrialization of materials in the future. Clearly, however, there is a limit to determining the individual peaks accurately just by retention time through the DAD-UV wavelength. On the other hand, more reliable data can be obtained using the MS detector. In this study, to compensate for these defects, verification experiments were conducted on five compounds detected through an analysis developed with HPLC/UV using one of the most powerful MS, UHPLC-QTOF/MS. In the same manner, further results of validation tests under the ICH Guide (International Conference on Harmonization) have become an important factor in verifying the developed HPLC/UV analysis method. In addition, validation studies including quantitative analysis of steroid compounds in DN through QTOF/MS analysis had already been reported [3], but the results of the recovery rate were found to be 72.79% to 118.31%, and showed significant differences in accuracy and recoveries when compared with the results of this study using UV detector.

Therefore, the photodiode array (PDA) spectrum (190–800 nm) was used to determine the maximum absorbance of each compound, and a wavelength of 200 nm was chosen as the optimum wavelength for analysis. Reviews of UV/VIS analyses of several previously reported protodioscins have shown that the maximum absorbance of the compound is approximately 1.6 times greater at 200 nm [11], although a number of studies primarily selected wavelengths of 205 nm [8,24,25], possibly to reduce the effect of baseline signals from solvents or impurities. However, using a wavelength of 200 nm, the limit of detection (LOD) and limit of quantitation (LOQ) were determined by analyzing standards at various concentrations to evaluate whether this wavelength is suitable for developing an analysis method based on UV detection.

To optimize the conditions for analysis of steroidal saponins derived from *Dioscorea*, sufficient baseline separation between structurally similar compounds, between dioscin and gracillin and between protodioscin and protogracillin had to be achieved. Preliminary experiments were conducted using a variety of columns to improve peak resolution. Finally, these steroid glucoside compounds could be conveniently separated using a Triart C18 PFP resin column, which has lower polarity and higher hydrophobicity than a standard C18 column. Meanwhile, a previous study reported the optimal analysis conditions for compounds **1**, **2**, **4**, and **5** in DN and the resolutions of three different stationary phases (Hypersil GOLD C18, Unison UK-C18, and Kinetex C18 column) and temperatures (15 °C to 45 °C), and the Kinetex C18 column presented the most efficient separation when analysis was performed at 15 °C [26]. However, although the conditions in this previous study allowed more rapid analysis than those used in this study, the previous analysis did not include compound **3**.

**Figure 2:**
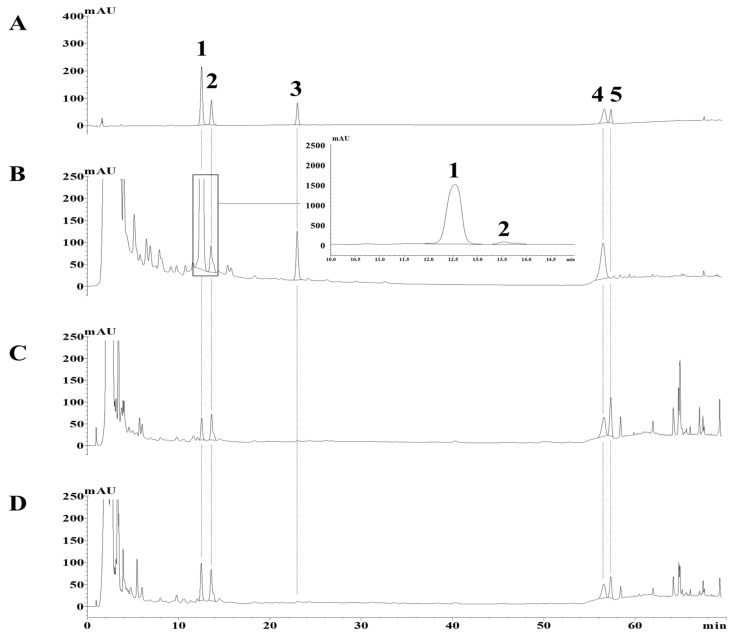
HPLC chromatograms of five standards (**A**, 1 mg/mL); and the DN (**B**, 40 mg/mL), DQ (**C**, 40 mg/mL) and DS (**D**, 40 mg/mL) samples. DN: *Dioscorea nipponica;* DQ: *Dioscorea quinquelobata*. DS: *Dioscorea septemloba*.

Furthermore, with the selected column, the sample was eluted under gradient conditions using a mixture of acetonitrile (A) and triple-distilled water (B). The compounds were not stable when a methanolic eluent was used, as previously reported [4,8,9], because protodioscin (furanol saponin) is rapidly converted to methyl protodioscin, its 22-*O*-methyl analogue, under high pH or methanol conditions. In terms of the solvent gradient program and column temperature, gradient elution with A/B = 23/77 to 33/67 (0–25 min), 33/67 to 34/66 (25–50 min), and 34/66 to 100/0 (5–65 min) at a flow rate of 1 mL/min and at 40 °C provided the optimal separation performance. Under these HPLC conditions, all compounds were detected without interference from impurities, and their retention times were 12.4 (**1**), 13.5 (**2**), 22.9 (**3**), 56.3 (**4**), and 57.2 (**5**) min (Figure 2). Further, based on the analysis results at 200 nm for the five types of compounds present in each sample (DN, DS, and DQ) under the conditions set above, UV spectra with a wavelength range of 190 to 800 nm were evaluated to examine the peaks for each compound according to retention time, and a total purity of 95% was verified.

### 2.2. Assay Validation

#### 2.2.1. Linearity

A linearity test was conducted to validate the calibration curve produced by the developed method. Between 7 and 12 sequentially diluted solutions of the test compounds at different concentrations (i.e., 0.01, 0.02, 0.03, 0.05, 0.1, 0.2, 0.3, 0.5, 0.7, 1, 2, and 4 mg/mL) were analyzed in triplicate. The calibration curves of the five tested compounds showed sufficient linearity with *r*^2^ values of 0.9992 (**1**), 0.9998 (**2**), 0.9995 (**3**), 0.9990 (**4**), and 0.9999 (**5**) (Table 1).

#### 2.2.2. Precision and Accuracy

The accuracy and precision were tested using two different samples, DN and DQ, because they contained different proportions of the target compounds. As shown in Figure 2, the chromatograph of DS was similar to that of DQ, and they had the same chemical component composition. Thus, DQ, which is more important, was used for subsequent accuracy and precision experiments.

To verify the accuracy of the analysis method, recovery experiments were conducted, and the average recoveries of the DN samples after being spiked with compounds **1**, **2**, **3**, and **4** at three different concentrations were found to be 92.1% to 100.9% (**1**), 100.0% to 100.6% (**2**), 100.2% to 100.3% (**3**), and 100.1% to 102.6% (**4**). In the case of DQ, the recoveries of compounds **1**, **2**, **4,** and **5** were found to be 93.7% to 101.0% (**1**), 98.3% to 106.2% (**2**), 96.4% to 100.8% (**4**), and 93.8% to 104.5% (**5**).

To assess the precision reliability of the analysis method, the RSD (relative standard deviation) values (%) of intra- and interday experiments were determined. In the intraday variability test, the RSD values for compounds **1**, **2**, **3**, and **4** in DN ranged from 0.03 to 0.05 (**1**), 0.14 to 0.22 (**2**), 0.04 to 0.16 (**3**), and 0.14 to 0.22 (**4**), and in DQ, the RSD values of compounds **1**, **2**, **4** and **5** were identified as 1.91 (**1**), 0.31 to 1.15 (**2**), 0.26 to 0.44 (**4**), and 0.27 to 0.82 (**5**). The RSD (%) of interday analyses conducted on three consecutive days for precision verification ranged from 0.01 to 0.01 (**1**), 0.12 to 0.01 (**2**), 0.03 to 0.10 (**3**), and 0.10 to 1.48 (**4**) for DN. As with accuracy, the precision was also verified for DQ, and the RSD (%) values of the compounds ranged between 0.15 and 0.55 (**1**), 0.48 and 0.73 (**2**), 0.38 and 0.75 (**4**), and 0.29 and 0.70 (**5**). These results indicate that the precision and accuracy of the method are sufficient to ensure that simultaneous analysis of the five compounds in DN and DQ is reliable and accurate (Table 2 and Table 3)

#### 2.2.3. Limit of Detection (LOD) and Quantification (LOQ)

The LOD and LOQ were calculated based on the standard deviation of the response and the slope of the calibration curve. Thus, the average SD value (*n* = 5) obtained by analyzing each of the five low-concentration compounds was substituted for the regression curve obtained from at least five low-concentration solutions, each of which was diluted sequentially as follows: 0.0005 ~ 0.005 mg/mL for 1, 0.001 ~ 0.01 mg/mL for 2 and 3, and 0.005 ~ 0.02 mg/mL for 4 and 5. From these results, the LOD was calculated by multiplying the LOD by 3.3 and the LOQ by 10. As a result, the LOD was determined to be 0.0009 mg/mL for compound **1**, 0.0022 mg/mL for compound **2**, 0.0007 mg/mL for compound **3**, 0.0132 for compound **4**, and 0.0027 for compound **5**. Previous studies detected protodioscin (**1**) at values up to approximately 0.0016 or 0.0039 mg/mL (LOD) [27,28], and these research results also showed that the LOD was 0.0009 mg/mL, although there was a difference in the analysis wavelength. The above results show that the analysis method developed for steroidal saponins provides sufficient sensitivity, likely due to mechanical and engineering advances in analytical systems and detectors (Table 1).

### 2.3. Quantitation of Compounds **1**–**5**

Using the developed analysis method, the reproducibility and accuracy of which were confirmed through HPLC validation, protodioscin (**1**), protogracillin (**2**), pseudoprotodioscin (**3**), dioscin (**4**), and gracillin (**5**) were successfully determined in DN, DQ, and DS. In DN, except for gracillin (**5**), the quantities of the four compounds tested were confirmed as 159.983 ± 0.064 mg/g for **1**, 4.250 ± 0.024 mg/g for **2**, 13.821 ± 0.037 mg/g for **3**, and 22.999 ± 0.121 mg/g for **4**. Pseudoprotodioscin (**3**) was not detected in DQ or DS, and the other compounds, protodioscin (**1**), protogracillin (**2**), dioscin (**4**), and gracillin (**5**), were found in DQ at levels of 3.496 ± 0.018 mg/g for **1**, 5.945 ± 0.020 mg/g for **2**, 10.002 ± 0.051 mg/g for **4**, and 9.011 ± 0.098 mg/g for **5**. In DS, the content of **1**, **2**, **4**, and **5** was 8.959 ± 0.014 mg/g, 9.902 ± 0.061 mg/g, 9.822 ± 0.014 mg/g, and 7.123 ± 0.031 mg/g, respectively. To summarize the above results, DN, DQ, and DS can be confirmed as the plants of origin based on the presence of pseudoprotodioscin and gracillin, and in the case of DN, the content of protodioscin is exceptionally high; thus, this component is expected to play a very important role in future activity and toxicity studies (Table 4).

### 2.4. Identification of Compound **1**–**5** in DN and DQ Using UHPLC-QTOF/MS

**Figure 3:**
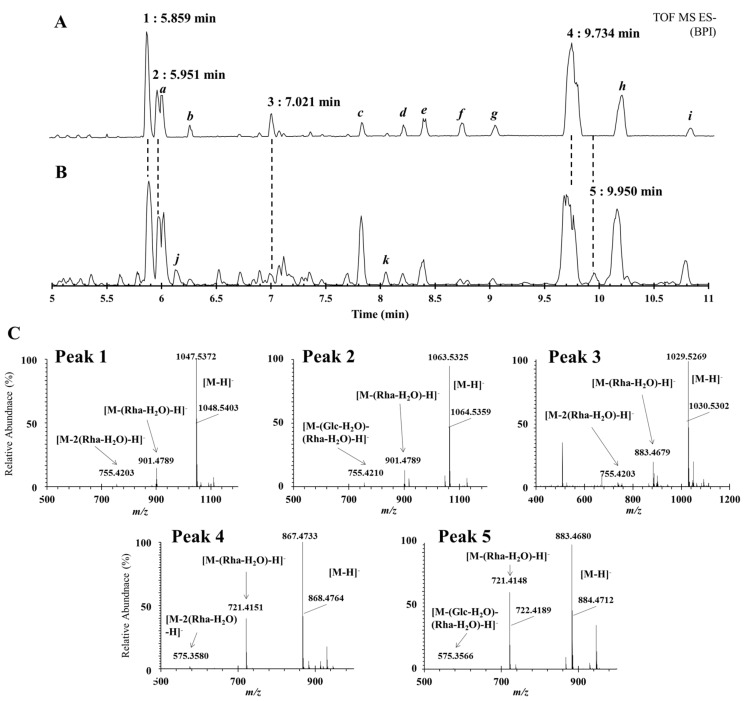
(**A**): Representative base peak intensity (BPI) chromatogram of DN and (**B**): DQ. (**C**): MS/MS spectrum of peak **1** (5.859 min), peak **2** (5.859 min), peak **3** (5.859 min), peak **4** (5.859 min), and peak **5** (5.859 min).

In the above experiments, using the developed method, a quantitative analysis of five compounds from DN and DQ was performed. However, to more accurately identify the compounds in the samples, a qualitative analysis using UHPLC-QTOF/MS was performed next. The total ion chromatograms (TICs) of DN and DQ were obtained, as shown in Figure 3, and compounds **1**, **2**, **3,** and **4** in DN and **1**, **2**, **4,** and **5** in DQ were observed, similar to the HPLC analysis results. In addition, based on the molecular formula of each compound, their MS/MS fragmentation data were compared, and their structures were inferred from these data. The retention times of the detected peaks were then checked, and the compounds were found to have lost two rhamnose moieties at ^1^Glc→^2^Rha and ^1^Glc→^4^Rha based on the MS/MS spectrum of the peak at 5.859 min (peak **1**), and these signals were identified as *m/z* 901.4789 [M-(rhamnose-H_2_O)-H]^-^ and 755.4203 [M-2(rhamnose-H_2_O)-H]^-^. Similar to peak **1**, peaks **3** and **4**, which were observed at 7.021 and 9.950 min, were also found to have lost two rhamnose moieties in the MS/MS spectrum; peak **3** showed *m/z* values of 883.4679 [M-(rhamnose-H_2_O)-H]^-^ and 737.4108 [M-2(rhamnose-H_2_O)-H]^-^, while peak **4** showed *m/z* values of 721.4151 [M-(rhamnose-H_2_O)-H]^-^ and 575.3580 [M-2(rhamnose-H_2_O)-H]^-^. On the other hand, unlike peaks **1**, **3,** and **4**, peaks **2** and **5** showed losses of glucose (^1^Glc→^3^Glc) and rhamnose (^1^Glc→^2^Rha). The MS/MS data showed fragment ions of 901.4789 [M-(glucose-H_2_O)-H]^-^ and 755.4210 *m/z* [M-(glucose-H_2_O)-(rhamnose-H_2_O)-H]^-^ for peak **2** and 721.4148 [M-(glucose-H_2_O)-H]^-^ and 575.3566 *m/z* [M-(glucose-H_2_O)-(rhamnose-H_2_O)-H]^-^ for peak **5**. Therefore, based on the above data and the existing literature [3,9], the structures of compounds **1**, **2**, **3**, **4,** and **5** in DN and DQ were confirmed as protodioscin (**1**), protogracillin (**2**), pseudoprotodioscin (**3**), dioscin (**4**), and gracillin (**5**) (Figure 3).

Meanwhile, the unidentified peaks (*a* to *k*) detected in the TCIs in Figure 3 were derived from the mass values of the parent molecule and fragmentation obtained through the auto MS/MS mode analysis using the Waters UNIFI software (Waters, Milford, MA, USA), and they are believed to be the most suitable compounds stored in the database library (Table 5).

Among these, *h*, the largest peak, was estimated to represent ophiopogonin B, a glycoside of the spirostane type found in *Dioscorea tokoro*, and it has a molecular weight of 722.4237 [29]. Additionally, a comparison of the ms/ms values of the product ions in the literature [30] showed values of 573.36 and 145.05 *m/z*, including 721.42 *m/z* [M-H]^−^. The peak *d* is steroid saponin, with a molecular weight of 884.4736, and compared with the MS value in the literature, we identified ion fragments of 883.47 and 737.41 *m/z*, which were estimated to represent spiroconazole A, which was isolated from *Dioscorea bulbifera* [31]. Similarly, peak *e* was estimated to be a compound reported as 2,7,2’- trihydroxy-4,4’,7’-trimethoxy-1,1’-biphenanthrene, derived from the leaves of DN [32]. Peak *g* was predicted as trillin, and it has primarily been reported in *Dioscorea zingiberensis*, but was also isolated from DN [33]. Additionally, prosapogenin A of dioscin (peak *i*) was previously detected in *Dioscorea zingiberensis* using QTOF/MS [34].

On the other hand, polyphylin V has been previously detected in DN using UPLC–qTOF–MS [3], although this study identified a similar derivative, polyphylin D, at peak *a*. Peak *f* was also predicted to be blumenol C glucoside, a component related to blumenol A, which has been previously isolated from DN [35]. Peak *k* was predicted to be neohecogenin-3-*O*-*β*-d-glucopyranoside, which is not a component reported in *Dioscorea* families, although hecogenin, which is dissociated from the sugar, was long ago reported as a major substance in *Dioscorea bernoulliana* [36].

The remaining peaks *b*, *c,* and *j* are considered to represent components that have not been discovered or reported thus far in *Dioscorea* families, including DN. These remaining unidentified peaks were carefully and tentatively identified, although the findings cannot be verified, using their MS/MS data as a reference. As a result, peaks *b* and *c* were identified as Timosaponin AIII [37] and Sanleng acid [38], respectively, based on the literature. Although the identity of the compound responsible for peak *j* at 6.14 min could not be inferred from the literature, it was presumed to be mutongsaponin C or akebia saponin F, according to the library program.

### 2.5. Anti-Osteosarcoma Effects of the Samples

Saponins demonstrate various pharmacological effects that can improve blood circulation, immune control, and antiviral effects. In addition, recent studies have reported that steroidal saponins exert effective anticancer activities, such as anti-invasion, anti-metastasis, and anti-angiogenic effects through various mechanisms, such as inhibition of cell proliferation and promotion of cell differentiation.

**Figure 4:**
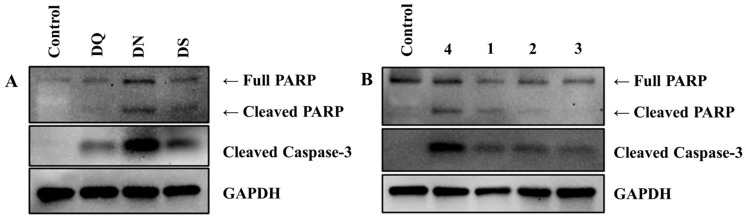
Effectiveness of DN against U2OS osteosarcoma cells. (**A**): U2OS cells treated with vehicle or 100 µg/mL extracts for 48 h. Apoptosis indexes (Cleaved Caspase-3 and PARP) were detected via western blotting. GAPDH served as the internal control. (**B**): U2OS cells treated with vehicle or 5 µM of each compound for 24 h.

Based on the characteristics of the samples analyzed above, the biological effects of DN, DQ, and DS were investigated. The steroidal saponins present in each sample exert their potential anticancer effects by activating apoptotic signaling. In particular, DN might have a stronger apoptotic effect because it has a higher steroidal saponins content relative to the other two extracts. Since these compounds are reported to have various pharmacological activities, such as anti-obesity activity [8], anti-inflammatory activity [39], protective effects against hyperlipidemia and oxidative stress [36], and anti-tumor activity [40], information on the composition and content of the steroidal saponin present in herbal medicines is important for future research. To verify their anticancer effects, a western blotting assay to confirm the expression levels of the apoptosis markers cleaved-Cas3 and cleaved-PARP in U2OS osteosarcoma cells was attempted. As expected, DN had a more powerful apoptosis-inducing effect than DQ and DS. Furthermore, to find the individual compound responsible for the pro-apoptotic effect of DN, we examined the levels of the apoptotic markers in the presence of the four major compounds, **1**, **2**, **3**, and **4**. The IC_50_ values of **1** and **4** were 6.43 µM and 6.90 µM, respectively, while those of **3** and **4** were 10.84 µM and 10.61 µM, respectively. Based on these results, we predicted that protodioscin (**1**) and dioscin (**4**) could be important in the mechanism underlying the pro-apoptosis effect of DN against osteosarcoma cells. To verify this hypothesis, the levels of apoptosis markers were evaluated after treatment of U2OS cells with the individual compounds at the same concentrations. Interestingly, **4** showed the most potent anticancer effect, and the other compounds were less effective than **4**. Dioscin (**4**) has already been shown to inhibit the growth of colon, ovarian, and lung cancer and to be effective in apoptosis of cancer cells [41,42,43]. The anticancer effect caused by **4** examines whether the anticancer effect of osteosarcoma also uses the mitochondria signal pathway. Thus, we would like to emphasize the importance of natural medicine in that the strong anticancer effect of **4** can be obtained from DN. Taken together, the powerful anticancer effect of DN can be attributed to its high dioscin (**4**) content, suggesting that it might be useful for osteosarcoma treatment (Figure 4).

## 3. Experimental

### 3.1. Reagents and Standards

Analytical-grade acetonitrile (ACN) and triple-distilled water were purchased from J. T. Baker (Philipsburg, NJ, USA). Standards of protodioscin (**1**), protogracillin (**2**), pseudoprotodioscin (**3**), dioscin (**4**), and gracillin (**5**) were purchased from ChemFaces (Wuhan, China), and these compounds (1–5) had purities of ≥98%. Stock solutions of the five reference compounds were prepared at concentrations of 5 mg/mL in 70% ACN and 30% water. The working solutions were prepared by serial dilution with 70% ACN to obtain a final concentration of 0.0005 mg/mL, and the solutions were stored at 4 °C until analysis.

### 3.2. Sample Preparation and Extraction

To secure the standard samples, experts from the Korea Institute of Oriental Medicine were consulted three times on collection of the samples. Ten different batches of DN raw material were collected from the regions of Eumseong and Yeongwol counties (Chungcheongbuk-do and Gangwon-do, Korea) in July 2018. Ten different batches of DQ and DS were collected from the regions of Aewol-eup (Jeju Island, Korea) in June 2018. The plant materials were identified again by an inspection committee of herbal medicines from the Korea Institute of Oriental Medicine (KIOM). Furthermore, a portion of all the collected samples were subjected to genetic DNA sequencing and HPLC analysis, and the 10 samples in each of the three groups were confirmed to be genetically and chemically identical to the other group members (data not shown). Then, all the raw materials and extracts were deposited at the KIOM (KIOM R 1803051-1 to 5).

Samples from all three groups were cleaned and dried at 50 °C for seven days in a drying oven. The dried samples were powdered, and 300 g of each sample was extracted twice for three hours using 2 L of 70% ethanol at 70 °C via reflux extraction. The extracts of the samples were concentrated at 40 °C to approximately one-quarter of their initial volume using a vacuum evaporator. Ethanol was removed from the residues by freeze-drying for 7 days, and the residual material was used for subsequent experiments.

To prepare samples for HPLC analysis, 40 mg of the extracted and dried material was dissolved in 2 mL of 70% ACN by sonication for 10 min. After centrifugation for 10 min at 4000× *g*, the supernatant was filtered through a disposable syringe filter (0.22 µm, 25 mm, CA syringe filter), obtained from Futecs Co., Ltd. (Daejeon, Korea), prior to injection into the HPLC system. The chromatographic peaks of the sample solution were identified by comparing their retention times and PDA spectra with those of standards.

### 3.3. HPLC Conditions

The HPLC analysis was conducted with a Shimadzu LC-20A Prominence Series system (Shimadzu Corporation, Kyoto, Japan) equipped with a quaternary pump (LC-20AD), vacuum degasser (DGU-20A3R), autosampler (SIL-20A), column oven (CTO-20A), and photodiode-array detector (SPD-M20A). Chromatographic data were interpreted using LabSolutions Multi PDA software. Chromatographic separation was performed on a YMC Triart C18 PFP column (4.6 × 250 mm i.d., 5 μm). The column oven was maintained at 40 °C, detection was conducted at *λ* = 200 nm, and online UV absorption spectra were recorded in a range from 190 to 400 nm. A gradient elution system was implemented as follows: mobile phase A, acetonitrile/B, triple-distilled water = 23/77 to 33/67 (0–25 min), 33/67 to 34/66 (25–50 min), and 34/66 to 100/0 (50–65 min) at a 1 mL/min flow rate at 40 °C, with an injection volume of 10 μL.

### 3.4. Validation Method for HPLC

The HPLC analysis method was validated in terms of linearity, accuracy, precision, LOD, and LOQ following the guidelines set by the International Conference on Harmonization (ICH). The linearity was established by evaluating the value of *r*^2^ (correlation coefficient) for the calibration curve prepared from ten serially diluted solutions. The precision of the analysis method was examined using the intermediate evaluation method by measuring the intra- and interday variability. The intraday variability was determined by analyzing the sample solution on one of the study days (24 h), while the interday variability was determined by injecting the sample solutions five times per day on four different days. The relative standard deviation (RSD) values were calculated for both the retention time and peak area from these five experiments. The RSD is considered a measure of precision. Recovery tests using a sample solution spiked with each standard compound were performed to evaluate accuracy. Recovery rates were determined by calculating the mean recovery (%) of the standards from the spiked extract solutions vs. the recovery from the nonspiked extract. The LOD and LOQ were calculated based on the standard deviation of the response and the slope of the calibration curve; an S/N ratio of 3 was used for the LOD, and an S/N ratio of 10 was used for the LOQ.

### 3.5. UHPLC-QTOF/MS Analysis

An unbiased metabolomics analysis was performed using a UPLC system (Waters, Milford, USA). The chromatographic separation was carried out using an ACQUITY UPLC HSS T3 column (100 mm × 2.1 mm, 1.8 μm, Waters) with a column temperature of 40 °C and a flow rate of 0.5 mL/min. The mobile phase contained solvent A (water + 0.1% formic acid) and solvent B (acetonitrile + 0.1% formic acid). The metabolites were eluted using the following gradient elution conditions: 97% phase A for 0–1 min; 3%–100% linear gradient phase B for 5–16 min; 100% phase B for 16–17 min; 100%–3% reverse linear gradient phase B for 17–19 min; 97% phase A for 19–25 min. The injection volume was 5 μL. The metabolites in the eluate were detected with a high-resolution tandem mass spectrometer (SYNAPT G2 Si HDMS QTOF, Waters) in positive and negative ion modes. In positive ion mode, the capillary voltage and the cone voltage were 2 kV and 40 V, respectively. In negative ion mode, they were 1 kV and 40 V, respectively. Centroid MS^E^ mode was used to collect the mass spectrometry data. The primary scan ranged from 50 to 1200 Da, and the scanning time was 0.2 s. All the parent ions were fragmented using 20–40 eV. All the fragment data were collected, and the time was 0.2 s. In the data acquisition process, the signal of leucine enkephalin (LE) was obtained every 3 s for real-time quality correction. For accurate mass acquisition, LE at a flow rate of 10 μL min^−1^ was used as a lock mass with a lock spray interface to monitor both positive ([M + H]^+^ = 556.2771) and negative ([M − H]^−^ = 554.2615) ion modes. Data acquisition and analysis were controlled by Waters UNIFI V1.71 software. The MS and MS/MS scanning ranges were 50–1200 *m/z*.

### 3.6. Cell Culture

Human osteosarcoma U2OS cells were obtained from American Type Culture Collection (ATCC). The U2OS cells were cultured in Dulbecco’s modified Eagle’s medium (DMEM; Gibco, Grand Island, NY, USA) supplemented with 10% fetal bovine serum (FBS) and 1% penicillin/streptomycin at 37 °C in a 5% CO_2_ incubator.

### 3.7. Western Blot Analysis

Western blotting was performed according to the manufacturer’s instructions. Antibodies against PARP (1:1000, #9542, Cell Signaling), cleaved caspase-3 (1:1000, #9661, Cell Signaling), and GAPDH (1:3000, #5174, Cell Signaling) were used.

### 3.8. Cell Viability Assay

For cell viability assays, DN, DQ, and DS extract powders were dissolved in distilled water and then filtered through a membrane with a pore size of 0.2 µm. Each of the individual component compounds were dissolved separately in dimethyl sulfoxide and then filtered through the 0.2 µm membrane. A total of 1 × 10^3^ cells were plated in 96-well plates and exposed to the extracts (DN, DQ and DS) and individual compounds (protodioscin, dioscin, pseudoprotodioscin, and protogracillin) at different concentrations at a final volume of 100 µL. After 48 h, MTS (Promega) solution was added to the wells at 10 µL/well. The cells were incubated for an additional 1 h, and then, the absorbance at 490 nm was recorded with a 96-well plate reader.

## 4. Conclusions

In this study, a method was developed to chemically distinguish five steroidal saponins, protodioscin (1), protogracillin (2), pseudoprotodioscin (3), dioscin (4), and gracillin (5), using HPLC-UV analysis, and this method could be used to distinguish DN, which is used as an Oriental medicine, from DQ and related species. The HPLC analysis method developed above was validated based on linearity (*r*^2^ > 0.999), accuracy (92.1%–106.2%), precision (<1.91%), LOD (<0.0132 mg/mL), and LOQ (<0.04 mg/mL). In addition, a UHPLC-QTOF/MS analysis confirmed that the five steroidal saponins quantified by HPLC-UV were present in the samples, and their structures were predicted based on the loss of one or two sugars (rhamnose or glucose fragment ions), which were visible in the MS/MS spectra. Above all, the UHPLC-QTOF/MS results showed that the composition of the compounds in each of the different DN samples (1, 2, 3, and 4) and in each of the DQ samples (1, 2, 4, and 5) was consistent based on the HPLC-UV data. To the best of our knowledge, this method, which is based on five compounds, is the most accurate and straightforward system developed to date to identify DN. In addition, unlike many previous studies, DQ and DS, which are genetically and morphologically similar, can be compared using the approach developed here, and this information is important for future research or commercial development. Finally, based on the results of the anti-osteosarcoma activity tests and quantitative analysis of DN and its compounds, 1 and 4 were more abundant in DN than in DQ or DS, endowing DN with the strongest activity. Therefore, the strong activity of DN was due to the presence of protodioscin (1) and dioscin (4). We concluded that DN, which has been correctly determined through the above analyses, is a powerful and useful anti-osteosarcoma herbal remedy, and we are in the process of conducting a mechanistic study and in vivo experiments to further elucidate its pharmacological activity.

## Figures and Tables

**Table 1:** Linearity, LOD, and LOQ of the standard compounds.

Compounds	t*_R_* (min)	Equation (Linear Model)*^a^*	Linear Range (mg/mL)	*r^2 b^*	LOD *^c^* (mg/mL)	LOQ *^d^* (mg/mL)
**1**	12.4	y = 3,442,356x − 8492	0.02–4	0.9992	0.0009	0.0026
**2**	13.5	y = 1,353,127x + 13,963	0.02–4	0.9998	0.0022	0.0065
**3**	22.9	y = 1,269,657x – 23,913	0.02–4	0.9995	0.0007	0.0020
**4**	56.3	y = 1,528,845x + 2521	0.03–0.7	0.9990	0.0132	0.0400
**5**	57.2	y = 1,462,227x – 826	0.01–0.3	0.9999	0.0027	0.0081

*^a^* y: peak area at 200 nm; x: standard concentration (mg/mL). *^b^ r*^2^: coefficient of determination with 7–12 indicated points in the calibration curves. *^c^* LOD: limit of detection; S/N = 3 (*n* = 5). *^d^* LOQ: limit of quantification; S/N = 10 (*n* = 5).

**Table 2:** Accuracy, intraday and interday precision of the standard compounds in DS.

Compound	Spiked Amount (mg/mL)	Content (mg/mL)		Recovery Test (%, *n* = 5)	Precision Test (*n* = 5)	
		Un-Spiked	Measured		Intra-Day RSD*^a^*(%)	Inter-Day RSD (%)
**1**	0.03	3.229654	3.227270	92.1	0.05	0.01
	0.1	3.299654	3.300564	100.9	0.03	0.01
	0.3	3.499654	3.502000	100.8	0.03	0.01
**2**	0.03	0.115008	0.115157	100.5	0.22	0.31
	0.1	0.185008	0.185613	100.6	0.17	0.23
	0.3	0.385008	0.384965	100.0	0.14	0.12
**3**	0.03	0.306423	0.306506	100.3	0.04	0.10
	0.1	0.376423	0.376691	100.3	0.08	0.06
	0.3	0.576423	0.576923	100.2	0.16	0.03
**4**	0.03	0.489984	0.490762	102.6	0.20	0.15
	0.1	0.559984	0.561044	101.1	0.14	1.48
	0.3	0.759984	0.760324	100.1	0.22	0.10

*^a^* RSD: relative standard deviation.

**Table 3:** Accuracy, intra- and interday precision of the standard compounds in DQ.

Compound	Spiked Amount (mg/mL)	Content (mg/mL)		Recovery Test (%, *n =* 5)	Precision Test (*n =* 5)	
		Un-Spiked	Measured		Intra-Day RSD*^a^*(%)	Inter-Day RSD (%)
**1**	0.012	0.081910	0.0820287	101.0	0.24	0.55
	0.04	0.109910	0.1073792	93.7	1.91	0.15
	0.12	0.189910	0.188932	99.2	0.62	0.28
**2**	0.012	0.130902	0.1316462	106.2	0.31	0.73
	0.04	0.158902	0.1582045	98.3	0.75	0.50
	0.12	0.238902	0.2368625	98.3	1.15	0.48
**4**	0.012	0.212048	0.2116199	96.4	0.41	0.38
	0.04	0.240048	0.2400817	100.1	0.44	0.75
	0.12	0.320048	0.3210118	100.8	0.26	0.52
**5**	0.012	0.192213	0.1918098	96.6	0.27	0.29
	0.04	0.220213	0.2220230	104.5	0.67	0.70
	0.12	0.300213	0.2928230	93.8	0.82	0.33

*^a^* RSD: relative standard deviation.

**Table 4:** Content of compounds in the three different samples (mg/g).

Compound	Content (*n* = 4)		
	DN	DQ	DS
**1**	159.983 ± 0.064 *^a^*	3.496 ± 0.018	8.959 ± 0.014
**2**	4.250 ± 0.024	5.945 ± 0.020	9.902 ± 0.061
**3**	13.821 ± 0.037	N.D.	N.D.
**4**	22.999 ± 0.121	10.002 ± 0.051	9.822 ± 0.014
**5**	N.D.	9.011 ± 0.098	7.123 ± 0.031

*^a^* Standard error (mg/g).

**Table 5:** Estimates of unidentified peaks based on the MS/MS database library.

Sample	Peak	*t*_R_ (min)	Observed (Neutral)	Observed (*m/z*)	Mass Error (ppm)	Tentative Identification
DN (**A**)	*a*	6.03	854.4643	899.4625 [+HCOO]	−2.3	Polyphyllin D
	*b*	6.25	740.4350	785.4332 [+HCOO]	0.3	Timosaponin AIII
	*c*	7.85	330.2406	329.2334 [-H]	0.1	Sanleng acid
	*d*	8.18	884.4736	929.4718 [+HCOO]	−3.6	Spiroconazole A
	*e*	8.39	492.1566	537.1548 [+HCOO]	−1.3	2,7,2’-Trihydroxy-4,4’,7’-trimethoxy-1,1’-biphenanthrene
	*f*	8.73	372.2139	417.2121 [+HCOO]	−2.1	Blumenol C glucoside
	*g*	9.02	576.3642	621.3624 [+HCOO]	−3.2	Trillin
	*h*	10.06	722.4237	767.4219 [+HCOO, -H]	−0.5	Ophiopogonin B
	*i*	10.76	722.4236	767.4218 [+HCOO]	−0.7	Prosapogenin A of dioscin
DQ (**B**)	*j*	6.14	1090.5550	1135.5530 [+HCOO, -H]	−1.2	Mutongsaponin C or Akebia saponin F
	*k*	8.03	592.3596	637.3578 [+HCOO]	−2.4	Neohecogenin-3-*O*-β-d-glucopyranoside
